# Identification of the *BrC2DP* gene family in Chinese Cabbage and functional analysis of the *BrC2DP56* gene in drought stress

**DOI:** 10.3389/fpls.2026.1839463

**Published:** 2026-05-08

**Authors:** Chuanhong Liu, Zihan Li, Shiyao Dong, Jiaqi Sun, Shuang Wang, Xiaoxia Li, Hexuan Wang, Ying Wang, Xiaohong Li, Danmei Liu

**Affiliations:** 1 College of Agriculture, Liaodong University, Dandong, China; 2 Animal Disease Prevention and Control Center of Longcheng District, Chaoyang, China

**Keywords:** C2 domain-containing proteins, Chinese cabbage, drought stress, functional validation, genome-wide identification

## Abstract

C2 domain-containing proteins (C2DPs) constitute a family of calcium-dependent regulators involved in membrane trafficking and signal transduction, playing critical roles in plant growth, development, and stress responses. However, this gene family has not yet been systematically characterized in Chinese cabbage. In this study, 157 *BrC2DP* members were identified and found to be unevenly distributed across 10 chromosomes, with notable gene clusters. Phylogenetic analysis classified them into 7 groups, and collinearity analysis revealed 112 duplicated gene pairs, indicating that segmental duplications contributed substantially to the expansion of this family. Notably, expression analysis under drought stress identified *BrC2DP56* as a significantly up-regulated gene. Subcellular localization showed that *BrC2DP56* was localized to the endoplasmic reticulum. Functional assays demonstrated that transient silencing of *BrC2DP56* enhanced drought tolerance, whereas its overexpression increased susceptibility to drought, suggesting that *BrC2DP56* negatively regulates drought stress responses in Chinese cabbage. This study provided a theoretical basis for exploring the drought response mechanism of the *BrC2DP* family.

## Introduction

Chinese cabbage (*Brassica rapa* L. ssp. *pekinensis*) is a core leafy vegetable of the *Brassicaceae* family. However, water scarcity and drought stress driven by climate change have become key constraints on its yield and quality, with moderate drought reducing yield by 30%-50% and inhibiting the accumulation of vitamin C, soluble sugars and other nutrients (Chen et al., 2024), while also inducing decreased photosynthetic efficiency, excessive reactive oxygen species accumulation and impaired root water absorption, leading to weak plant growth and loose heading to further threaten stable production efficiency (Park et al., 2025). Therefore, dissecting the functions of key drought resistance-related genes and identifying new molecular breeding targets are of critical significance for breeding drought-tolerant Chinese cabbage varieties.

As a universally distributed and highly conserved second messenger in plants, Ca²^+^ participates in core physiological processes including plant growth and development, hormone signal transduction, and adaptive responses to external environmental stimuli (Kudla et al., 2010), and acts as a pivotal signaling hub for plants to cope with various abiotic stresses. A growing number of studies have confirmed that Ca²^+^ signaling pathways play an irreplaceable role in plant drought stress responses, mediating the whole process from stress signal perception to downstream physiological and molecular defense activation (Li et al., 2025). The core regulatory mechanism is that upon sensing drought-induced osmotic stress, plant cells rapidly trigger transient elevation of cytosolic Ca²^+^ concentration; Ca²^+^ binds to specific Ca²^+^ -binding proteins and induces conformational changes of these sensors, which further transmit Ca²^+^ signals to downstream signaling cascades, promote the expression of drought-responsive functional and regulatory genes, and ultimately enhance plant drought resistance by regulating stomatal closure, osmotic adjustment, and reactive oxygen species scavenging.

As a class of evolutionarily highly conserved Ca²^+^-dependent functional proteins, C2 domain-containing proteins (C2DPs) are widely distributed in eukaryotes, with a typical C2 domain consisting of approximately 135 amino acids, which was originally annotated as PKC-C2 domain in the PFAM database (PF00168) and characterized as a key Ca²^+^-sensing and membrane-targeting module (Plant et al., 1997). Functional studies of C2DPs in plants have gradually expanded, revealing their diverse roles in growth regulation and stress responses beyond basic cellular processes. In *Arabidopsis thaliana*, the synaptotagmin family gene *SYT1* encodes a C2DP that maintains plasma membrane integrity and ensures cell viability under adverse conditions, while the QKY gene (a typical multiple C2 domain and transmembrane region protein, MCTP) containing four predicted C2 domains, with its mutant showing defective development of root hairs, floral meristems, stems, floral organs and outer integuments (Vaddepalli et al., 2014). Multiple rice *C2DPs* have been verified to mediate abiotic stress tolerance, including *OsSMCP1*, a single C2 domain-containing protein whose overexpression enhances plant resistance to drought, salt, osmotic and oxidative stresses, and *OsC2DP* (*LOC_Os09g39770*), which is critical for salt stress tolerance with its CRISPR/Cas9 knockout mutant exhibiting hypersensitivity to salt and showing similar cytoplasm-to-membrane translocation under stress as *OsERG1* (Kim et al., 2003; Yokotani et al., 2009). A total of 3080 *C2DPs* were identified across species from lower algae to higher plants, and *OsNTMC2T2.2* negatively regulates freezing tolerance in rice (Zhao et al., 2025). A total of 90 *C2DP* family members were identified in *Lotus corniculatus*, which were found to respond to hormones and various stress conditions (Yang et al., 2025). To date, the *C2DP* gene family has merely been characterized in a limited range of plant species, such as soybean (Sun et al., 2021), rice (Zhang et al., 2022) and Sorghum bicolor (Niu et al., 2022). Nevertheless, systematic investigations into this gene family are still lacking in Chinese cabbage.

In this study, a comprehensive genome-wide bioinformatic analysis was conducted on the *BrC2DP* gene family in Chinese cabbage. Meanwhile, key genes associated with drought response within this gene family were screened through expression pattern analysis under drought stress, and functional verification of the target genes was implemented via experimental techniques including subcellular localization, gene silencing and transient overexpression. This study lays a foundation for further dissecting the functional roles of *BrC2DP* genes in drought stress response regulation in Chinese cabbage.

## Materials and methods

### Identification and analysis of physicochemical properties of the *BrC2DP* gene family

The Chinese cabbage database (Brara_Chiifu_V3.5) employed in this study was sourced from http://brassicadb.cn. To identify members of the *BrC2DP* gene family in Chinese cabbage, the hidden Markov model (HMM) profile of the C2 domain (PF00168) was obtained from the PFAM database (v.34.0 http://pfam.xfam.org/) and used to search against the Chinese cabbage reference proteome, using an e-value threshold of < e^−5^. The filtered results were submitted to the SMART (http://smart.embl-heidelberg.de/) and InterPro (https://www.ebi.ac.uk/interpro/) databases for validation to identify the members of the *BrC2DP* gene family. Furthermore, ExPASy World-Wide Web server (http://www.expasy.org/tools/) was employed to characterize the physicochemical properties of the *BrC2DP* family proteins (Gasteiger et al., 2005).

### Chromosomal localization, evolutionary analysis, and synteny analysis of the *BrC2DP* gene family

Based on the genomic information from the Chinese cabbage database, the genes of the *BrC2DP* family were mapped onto chromosomes and visualized using the ‘Gene Location Visualize from Table’ function of TBtools. Multiple sequence alignment of the nucleotide sequences was performed using the ClustalW algorithm implemented in MEGA 6.06, with the following alignment parameters: a gap opening penalty of 15, a gap extension penalty of 6.66, an IUB DNA weight matrix, and a transition weight of 0.5. Phylogenetic trees were constructed using the neighbor-joining (NJ) method, with the p-distance nucleotide substitution model applied to calculate pairwise genetic distances, incorporating both transitions and transversions and assuming uniform substitution rates across all nucleotide sites. Gaps and missing data in the aligned sequences were processed using the complete deletion strategy to exclude gap-containing positions from downstream analyses, and the statistical reliability of the resulting tree topology was assessed via the bootstrap method with 1000 bootstrap replicates. Finally, intra-species collinearity analysis was conducted with the ‘One Step MCScanX’ tool in TBtools to elucidate the evolution and conservation of this gene family. All analyses were performed using TBtools (Chen et al., 2023).

### Analysis of gene structure and motifs in the *BrC2DP* gene family

Gene structure was analyzed by extracting the coding sequences (CDS) and corresponding genomic sequences of the identified *BrC2DP* members, and the exon-intron organization was visualized using the ‘Gene Structure View (Advanced)’ tool in TBtools. For conserved motif analysis, the full-length protein sequences were submitted to the online MEME suite to identify 10 potential motifs with default settings, and the distribution of these discovered motifs was graphically presented using the ‘Visualize MEME/JASPAR Output’ function integrated within TBtools.

### Plant materials and drought treatment

The double haploid line ‘FT’ of Chinese cabbage was used as the experimental material. Sterile perlite, vermiculite and nutrient soil were thoroughly mixed at a 1:1:2 volume ratio to prepare the planting substrate. Seeds were sown in the substrate and cultivated in a growth chamber with a photoperiod of 16 h light/8 h dark and a constant temperature of 25 °C.

Uniform seedlings at the four-leaf stage with one heart leaf were selected and randomly divided into a control group (CK) and drought stress groups (D2, D4, D6), with three biological replicates per group. For the control group, the irrigation regime and culture conditions remained consistent with those before stress initiation, and no drought treatment was imposed throughout the experiment. For the drought stress groups, irrigation was suspended, while other culture conditions (light, temperature, and humidity) were maintained identical to the control group. The seedlings were subjected to drought stress for 2, 4, and 6 days, respectively.

### Subcellular localization

The full-length cDNA of *BrC2DP56* was amplified by PCR and cloned into the CaMV 35S promoter-driven pCAMV35S-GFP fluorescent vector to generate the BrC2DP56-GFP fusion construct, with the HDEL-mcherry endoplasmic reticulum marker vector prepared simultaneously. Both vectors were separately transformed into *Agrobacterium tumefaciens* strain GV3101. Positive colonies were cultured at 28 °C with shaking to an OD600 of 0.6–0.8, then mixed 1:1 and infiltrated into *Nicotiana benthamiana* leaves. Infiltrated plants were incubated in darkness for 24 h followed by 24 h light treatment, and leaf discs at the infiltration site were collected for slide preparation. Fluorescence signals were detected using a laser scanning confocal microscope (Leica Microsystems, Wetzlar, Germany): GFP fluorescence was monitored at 496–540 nm, paired with synchronous detection of HDEL-mcherry ER red fluorescence, and the ER subcellular localization of BrC2DP56 was determined based on fluorescent co-localization.

### Gene Silencing of *BrC2DP56* in Chinese cabbage

Virus-induced gene silencing (VIGS) mediated by tobacco rattle virus (TRV) was performed to characterize the function of *BrC2DP56* in Chinese cabbage. A 300 bp *BrC2DP56*-specific fragment was amplified and cloned into pTRV2 to generate the recombinant vector pTRV2-*BrC2DP56*, as described previously (Pflieger et al., 2008). Recombinant pTRV2-*BrC2DP56*, empty pTRV2 (negative control) and helper vector pTRV1 were verified by restriction digestion and sequencing, then separately transformed into *Agrobacterium tumefaciens* GV3101 (Zhou et al., 2021). Positive agrobacterium colonies were cultured and adjusted to OD_600_ = 0.8-1.0. The pTRV1 suspension was mixed with pTRV2 or pTRV2-*BrC2DP56* suspension at a 1:1 ratio, incubated at room temperature for 1 h, then used to infect newly germinated ‘FT’ seeds. Each treatment contained 50 seeds with three biological replicates. Infected seeds were sown in sterilized soil and grown in a growth chamber. *BrC2DP56* silencing efficiency was evaluated via gene expression analysis using leaf samples collected at 20 days post-infection.

### Transient overexpression analysis

The stop codon-free coding sequence of *BrC2DP56* was cloned into the pSuper1300-GFP vector to generate the recombinant overexpression construct, with the empty pSuper::GFP vector serving as the control. Both constructs were transiently transformed into germinated seeds of the ‘FT’ variety via *Agrobacterium* mediated agroinfiltration. For each treatment, 50 seeds were used with three biological replicates, and all seeds were cultivated in a growth chamber. Transient overexpression efficiency was evaluated at 20 days post agroinfiltration.

### qRT-PCR

Total RNA was extracted exclusively from leaf tissues of Chinese cabbage using a commercial RNA isolation kit (Vazyme, China). The tested samples were collected from four distinct plant groups: blank control plants, plants exposed to drought stress for 2, 4, and 6 days, gene-silenced plants, and *BrC2DP56* transient overexpression plants. Following RNA extraction, high-quality total RNA was reverse-transcribed into cDNA with the FastQuant RT Super Mix kit (TIANGEN, China), and all extraction and reverse transcription procedures were performed strictly in accordance with the corresponding kit instructions. The *Actin* gene was adopted as the endogenous reference gene to normalize target gene expression levels. Primer information is shown in **Supplementary Table 1**. Relative gene expression quantification was calculated using the 2^−ΔΔCt^ method.

### KEGG and GO enrichment analysis

The GO and KEGG enrichment analyses were performed using the Metascape online platform. Briefly, the gene symbols of the *BrC2DP* family were uploaded, and *Arabidopsis thaliana* was selected as the reference species. All analyses were conducted with default parameters, including a P-value cutoff ≤ 0.01, enrichment score≥1.5, and a minimum overlap of ≥3 genes. Upon completion of the analysis, the enrichment results were retrieved from the report page, covering three categories of Gene Ontology terms: biological process, cellular component, and molecular function, as well as the KEGG pathway enrichment data.

### Statistical analysis

All experiments were carried out with three independent biological replicates. Data were presented as mean ± standard error (SE) or mean ± standard deviation (SD). Statistical analyses were performed using GraphPad Prism 9.0.

An independent-samples t-test was used for analysis of significant differences between the two treatment groups. For comparisons among three or more treatment groups, one-way analysis of variance (one-way ANOVA) was conducted, followed by Duncan’s multiple range test for *post-hoc* multiple comparisons. Differences were regarded as statistically significant at P < 0.05 and highly significant at P < 0.01.

## Results

### Identification and physicochemical properties analysis of the *BrC2DP* gene family

A total of 157 *BrC2DP* gene family members were identified in Chinese cabbage, all of which contain the C2 domain (PF00168). The C2 domain in these candidate sequences was verified, and the 157 *BrC2DP* genes were systematically named *BrC2DP1* to *BrC2DP157* in accordance with their chromosomal positions from chromosome 1 to chromosome 10.

The BrC2DP proteins exhibited diverse fundamental characteristics. The number of amino acids ranged from 123 (BrC2DP2) to 2,756 (BrC2DP1). Their physicochemical properties also showed general variations. The molecular weight (MW) spanned from 13.8 (BrC2DP2) to 309.4 kDa (BrC2DP1), while the theoretical isoelectric point (pI) values ranged from 4.28 (BrC2DP121) to 9.95 (BrC2DP144). Among the BrC2DP family, 62 proteins were predicted to be stable with an instability index below 40, with the majority (95 proteins) being classified as unstable (**Supplementary Table 2**).

### Chromosomal locations and gene clusters of the *BrC2DP* gene family

**Figure 1:**
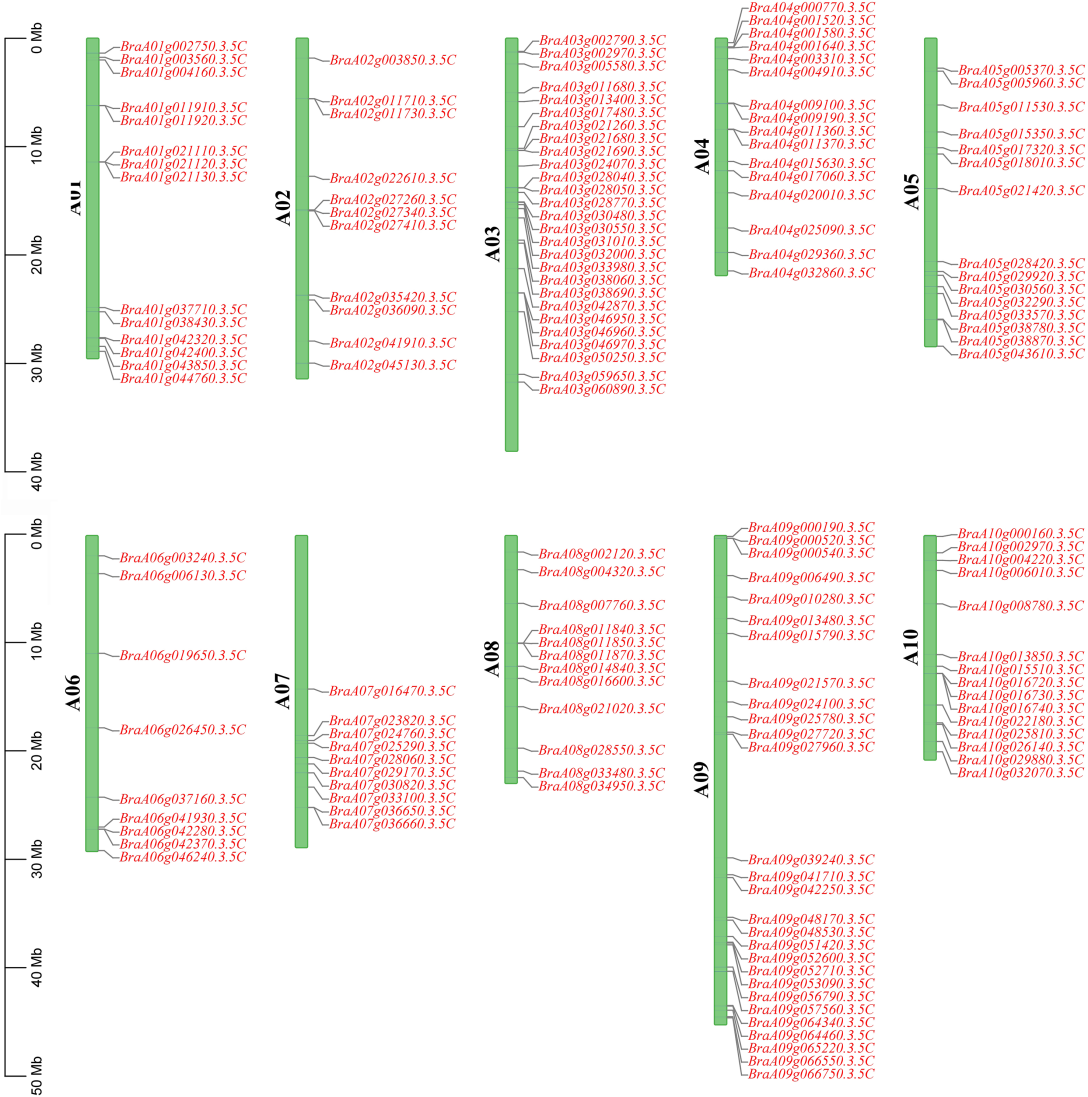
Chromosomal localization of the *BrC2DP* gene family in Chinese cabbage. Green rectangles represented chromosomes.

Extraction of chromosomal information of the *BrC2DP* gene family from the Chinese cabbage genome revealed that 157 *BrC2DP* genes were distributed on each of the 10 haploid chromosomes (n=10) (**Figure 1**). Among them, chromosome A06 contained the fewest *BrC2DP* genes, with 9 (*BrC2DP84*-*BrC2DP92*); chromosome A09 had the most *BrC2DP* genes, with 28 (*BrC2DP115*-*BrC2DP142*). The *BrC2DP* gene family exhibited a ‘Gene Cluster’ distribution pattern on some chromosomes, specifically as follows: Chromosome A01 had *BraA01g021110.3.5C*, *BraA01g021120.3.5C*, and *BraA01g021130.3.5C*; Chromosome A02 had *BraA02g027260.3.5C*, *BraA02g027340.3.5C*, and *BraA02g027410.3.5C*; Chromosome A03 had five gene clusters, including the first one with *BraA03g002790.3.5C*, *BraA03g002970.3.5C*, *BraA03g005580.3.5C*, the second one with *BraA03g021260.3.5C*, *BraA03g021680.3.5C*, *BraA03g021690.3.5C*, the third one with *BraA03g030480.3.5C*, *BraA03g030550.3.5C*, *BraA03g031010.3.5C*, *BraA03g032000.3.5C*, *BraA03g033980.3.5C*, the fourth one with *BraA03g046950.3.5C*, *BraA03g046960.3.5C*, *BraA03g046970.3.5C*; Chromosome A04 had *BraA04g000770.3.5C*, *BraA04g001520.3.5C*, *BraA04g001580.3.5C*, and *BraA04g001640.3.5C*; Chromosome A06 had *BraA06g041930.3.5C*, *BraA06g042280.3.5C*, *BraA06g042370.3.5C*; Chromosome A08 had *BraA08g011840.3.5C*, *BraA08g011850.3.5C*, and *BraA08g011870.3.5C*; Chromosome A09 had two gene clusters, with the first one containing *BraA09g000190.3.5C*, *BraA09g000520.3.5C*, *BraA09g000540.3.5C* and the second one containing *BraA09g052600.3.5C*, *BraA09g052710.3.5C*, *BraA09g053090.3.5C*; Chromosome A10 had *BraA10g016720.3.5C*, *BraA10g016730.3.5C*, and *BraA10g016740.3.5C*. Gene clusters were typically generated by gene duplication events, and the presence of multiple gene clusters in the *BrC2DP* gene family indicated that this gene family had undergone multiple independent duplication events throughout its evolutionary history.

### Evolutionary and collinearity analysis of the *BrC2DP* gene family

**Figure 2:**
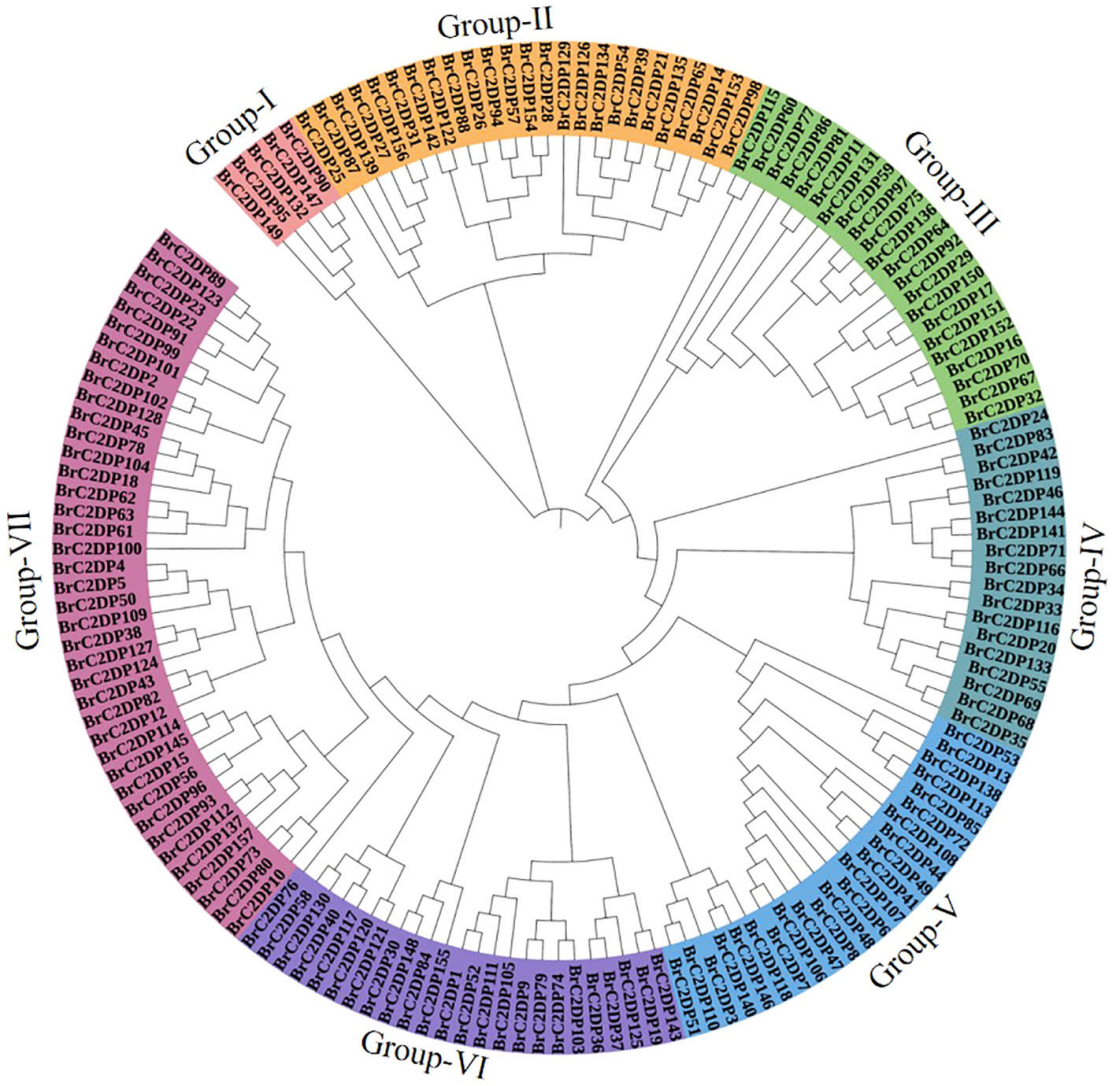
Evolutionary analysis of the *BrC2DP* gene family in Chinese cabbage. Different colors represented distinct groups.

Phylogenetic tree analysis clustered the Chinese cabbage *BrC2DP* gene family members into seven major groups (Group-I to Group-VII), indicated by different colors (**Figure 2**). Among these, Group-I contained the fewest genes, with only five members. This analysis revealed that the gene family underwent multiple duplication events during evolution, forming several subfamilies with high sequence homology.

**Figure 3:**
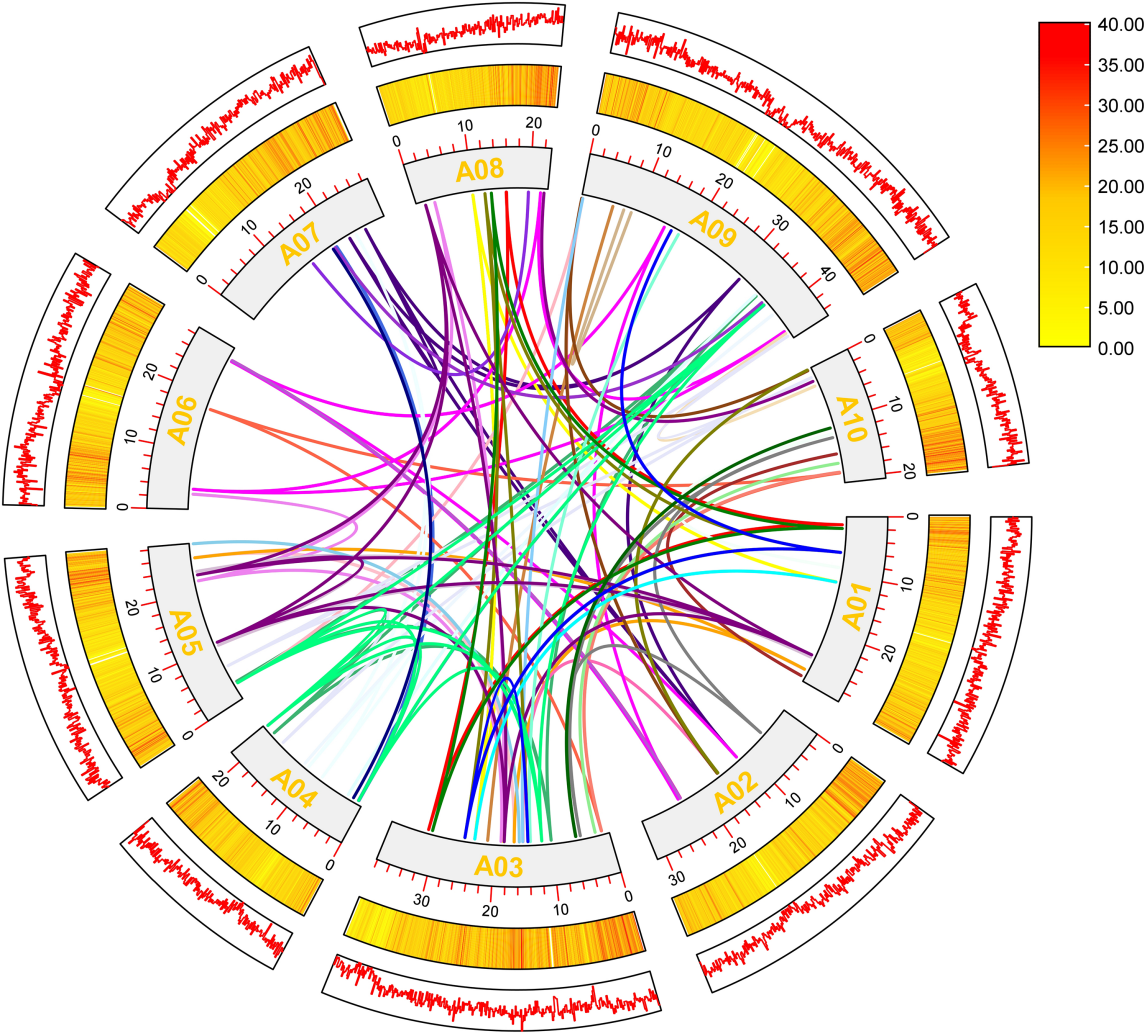
Synteny analysis of the *BrC2DP* gene family in Chinese cabbage.

Collinearity analysis of the *BrC2DP* gene family in Chinese cabbage revealed 112 collinear *BrC2DP* gene pairs distributed across chromosomes A01 to A10 (**Figure 3**). Chromosome A03 exhibited the highest level of involvement with 34 pairs, followed by chromosome A09 with 29 pairs, while chromosomes A06 (7 pairs) and A07 (10 pairs) showed the lowest level of involvement. The high activity on chromosomes A03 and A09 suggests that these regions may have undergone whole-genome duplication or segmental duplication events, promoting the expansion of the *BrC2DP* gene family.

### Gene structure and motifs of the Chinese cabbage *BrC2DP* gene family

**Figure 4:**
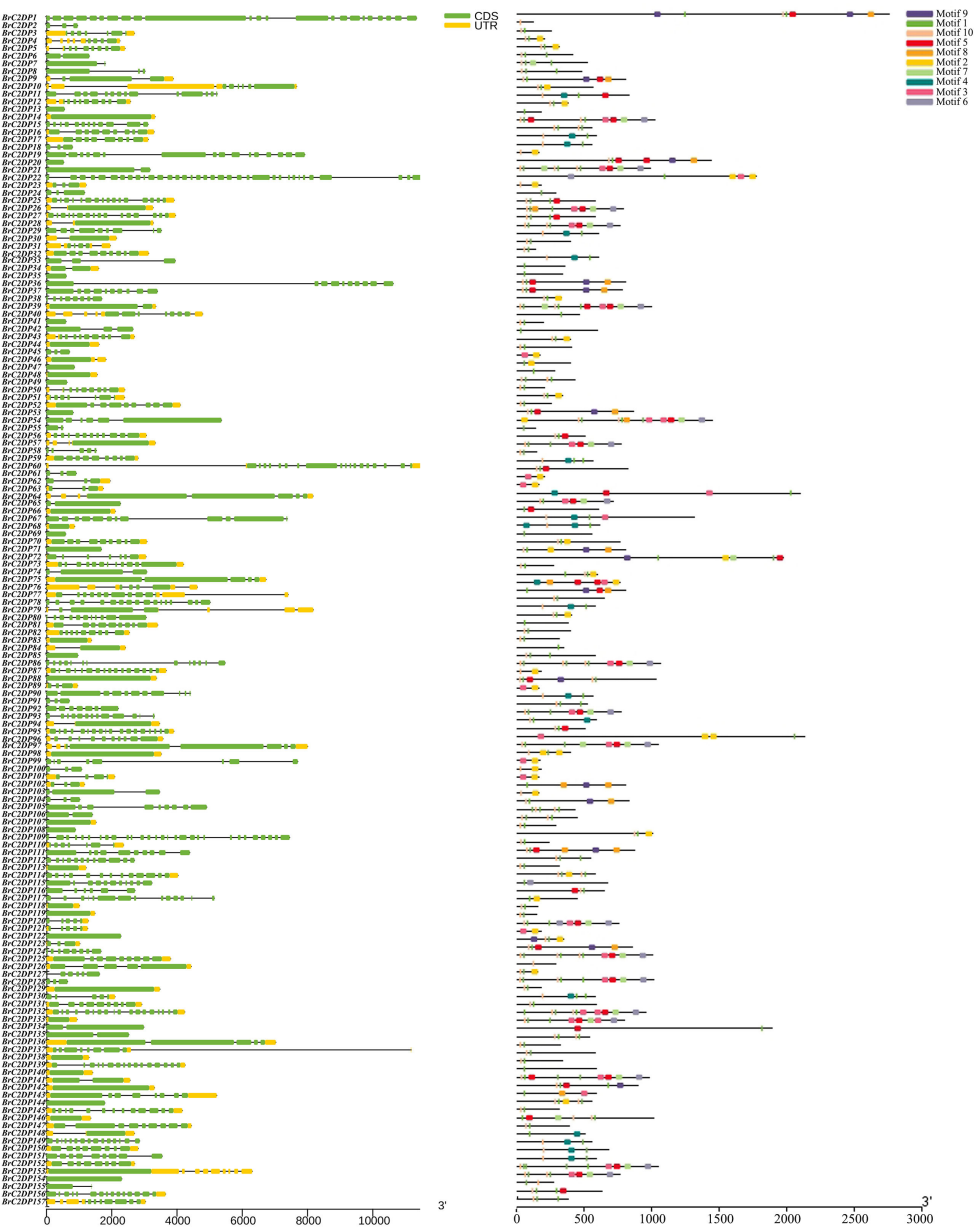
Gene structure and conserved motif analysis of the *BrC2DP* gene family in Chinese cabbage.

Statistical analysis of the exon numbers of 157 members in the Chinese cabbage *BrC2DP* gene family revealed a high degree of diversity in their gene structures (**Figure 4**). The number of exons varied widely, ranging from a minimum of 1 to a maximum of 35 (*BrC2DP22*), with an average of approximately 6.18 exons per gene and a median of 5. Among them, genes containing only one exon were the most abundant (42 in total, accounting for 26.75%), indicating a substantial number of structurally simple genes within this family. The vast majority of genes (over 56%) had exon numbers ranging from 1 to 5, suggesting an overall tendency toward structural simplicity. However, a small number of genes exhibited complex structures with more than 20 exons (3 in total), which may imply more intricate functions or diverse regulatory mechanisms. In summary, the *BrC2DP* gene family is predominantly composed of members with fewer exons, but it also retains a small number of structurally complex members with high exon counts.

Statistical analysis of the motif composition of the *BrC2DP* gene family in Chinese cabbage revealed a highly specific distribution of motifs among its members (**Figure 4**). Motif 1 (91.1% occurrence frequency) and Motif 10 (73.2%) were identified as the absolutely dominant core conserved motifs, forming the common foundation for the vast majority of genes. This suggests their potential crucial role in maintaining the basic structure and function of this gene family. The occurrence frequencies of the remaining motifs varied significantly, with Motif 5 (28.7%), Motif 2 (22.9%), and Motif 3 (21.7%) being relatively common, while Motifs 4, 6, 7, 8, and 9 all exhibited occurrence frequencies below 15%, classifying them as rare motifs. The number of motifs per gene varied considerably, with most genes containing 2 to 5 motifs, though 10 members contained only a single motif. In summary, while preserving the core structure, the *BrC2DP* gene family achieves functional diversity and specificity through the acquisition of different auxiliary motifs.

### Expression of the *BrC2DP* gene family under drought conditions

**Figure 5:**
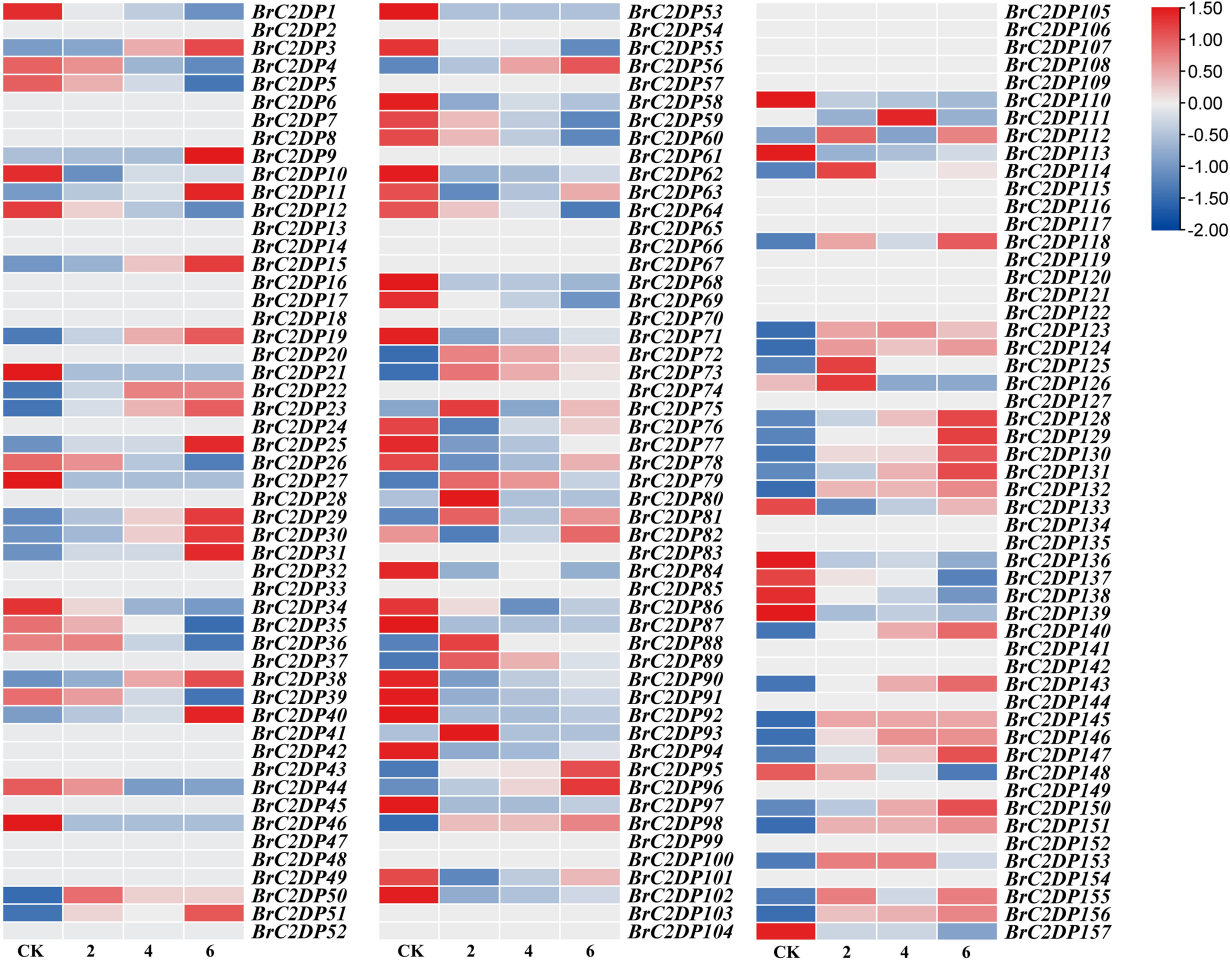
qRT-PCR analysis of the *BrC2DP* gene family in Chinese cabbage under drought stress.

To clarify the role of the *BrC2DP* gene family in drought stress in Chinese cabbage, the expression of the *BrC2DP* gene family was examined after 2, 4, and 6d of drought treatment. The results revealed that several *BrC2DP* genes exhibited differential expression in response to drought stress over time (**Figure 5**). There were 15 genes (*BrC2DP1*, *BrC2DP4*, *BrC2DP5*, *BrC2DP12*, *BrC2DP26*, *BrC2DP34*, *BrC2DP35*, *BrC2DP39*, *BrC2DP44*, *BrC2DP55*, *BrC2DP59*, *BrC2DP64*, *BrC2DP69*, *BrC2DP137*, *BrC2DP148*) that showed continuous downregulation, 17 genes (*BrC2DP3*, *BrC2DP11*, *BrC2DP19*, *BrC2DP23*, *BrC2DP29*, *BrC2DP30*, *BrC2DP38*, *BrC2DP56*, *BrC2DP95*, *BrC2DP96*, *BrC2DP128*, *BrC2DP131*, *BrC2DP140*, *BrC2DP143*, *BrC2DP147*, *BrC2DP150*, *BrC2DP156*) that showed continuous upregulation under the drought treatments conducted on 2, 4, and 6 d, which indicated that these genes might play an important role in drought stress response. Under drought stress conditions, the *BrC2DP56* gene exhibited the most significant up-regulation in expression, suggesting that it may serve as a central player in the *BrC2DP* family for drought stress response in Chinese cabbage. Based on this finding, the *BrC2DP56* gene was selected for further investigation into its potential role in conferring drought stress tolerance in Chinese cabbage.

### Subcellular localization of the BrC2DP56

**Figure 6:**
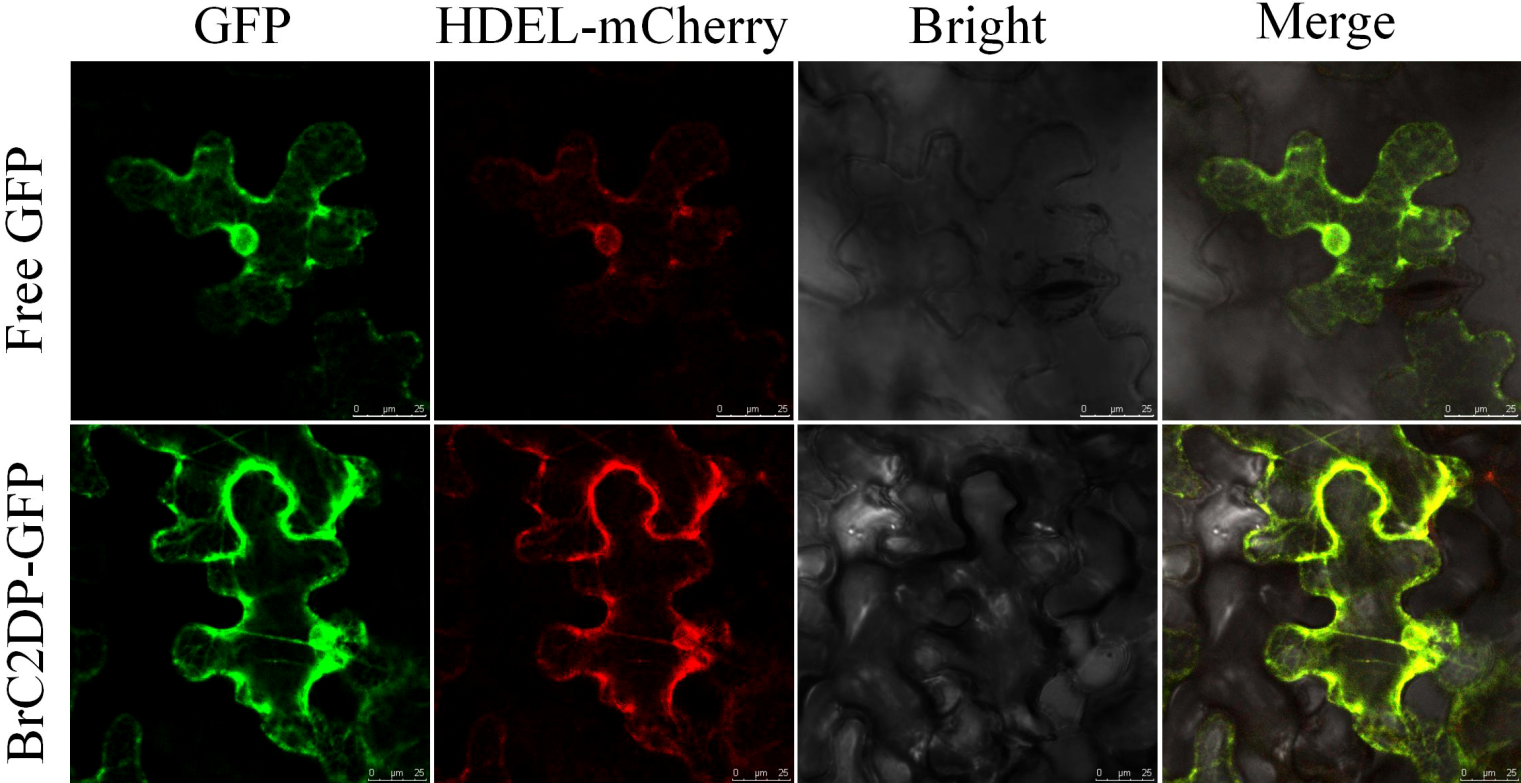
Subcellular localization of BrC2DP56.

To determine the subcellular localization of BrC2DP56 protein, the empty GFP vector and BrC2DP56-GFP fusion vector were transiently infiltrated into tobacco leaves. The endoplasmic reticulum-specific marker HDEL-mCherry was adopted as a co-localization reference, and fluorescence co-localization characteristics were detected via laser scanning confocal microscopy. Fluorescence observations showed that the green fluorescence derived from BrC2DP56-GFP completely overlapped with the red fluorescence of HDEL-mCherry, and both fluorescent signals were specifically distributed in the reticular structure of the endoplasmic reticulum (**Figure 6**). Taken together, the co-localization analysis confirmed that BrC2DP56 protein was localized to the endoplasmic reticulum.

### Response of *BrC2DP56* to drought stress

**Figure 7:**
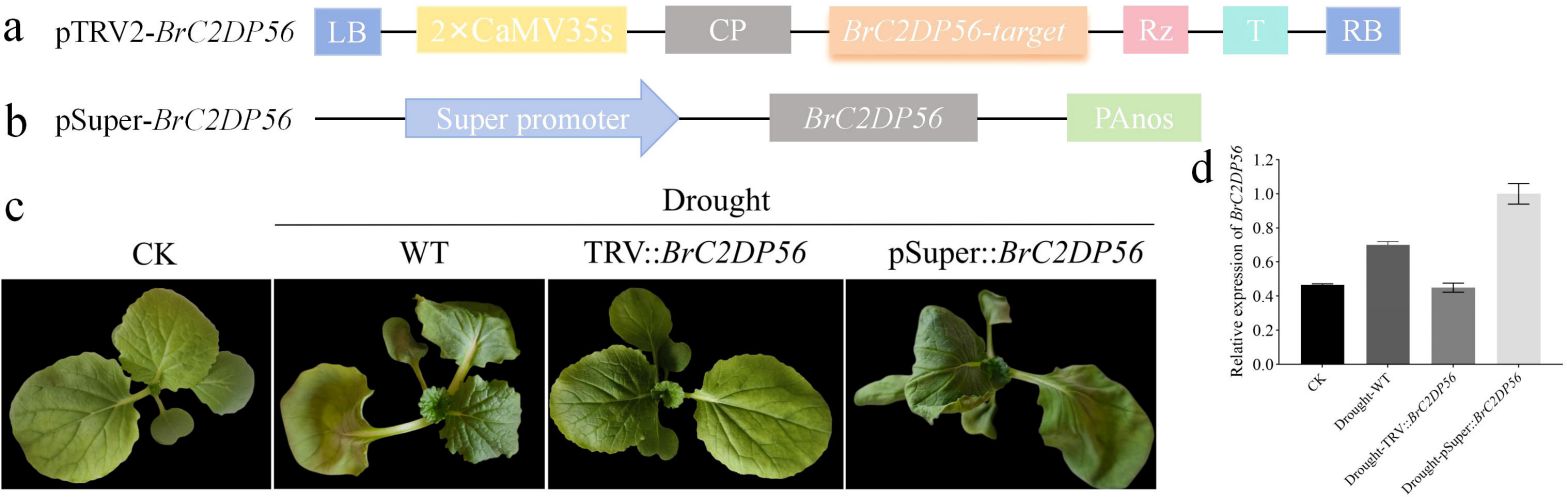
Functional verification of the *BrC2DP56* gene. **(A)** pTRV2 vector construction for *BrC2DP56*. **(B)** Transient overexpression vector structure of *BrC2DP56.* **(C)**. Phenotypes of *BrC2DP56* gene-silenced and transient overexpression plants under drought stress. **(D)** Expression level of *BrC2DP56* in gene−silenced and transient overexpression plants under drought stress.

To further clarify the function of *BrC2DP56* in responding to drought stress in Chinese cabbage, transient silencing and overexpression experiments were performed on ‘FT’. Subsequently, the plant phenotypes and the expression level of *BrC2DP56* were detected after 5 d of drought treatment. The pTRV2-*BrC2DP56* (**Figure 7a**) and pSuper::*BrC2DP56* (**Figure 7b**) vectors were constructed separately and then individually transformed into ‘FT’. After 5 d of drought stress, wild-type (WT) plant showed slight wilting (**Figure 7c**), with significantly upregulated *BrC2DP56* expression compared to non-drought-stressed control plants (**Figure 7d**). In contrast, *BrC2DP56* transient silencing plants (TRV::*BrC2DP56*) exhibited no obvious wilting (**Figure 7c**), and their *BrC2DP56* expression was significantly lower than that in WT (**Figure 7d**). Conversely, *BrC2DP56* transient overexpression plants (pSuper::*BrC2DP56*) displayed severe wilting (**Figure 7c**), accompanied by a significant increase in *BrC2DP56* expression relative to WT (**Figure 7d**). These results indicated that *BrC2DP56* expression is closely associated with drought stress tolerance in Chinese cabbage. Its overexpression exacerbated drought-induced wilting, while its suppression enhanced plant drought tolerance.

### KEGG and GO enrichment analysis of *BrC2DP* family genes

**Figure 8:**
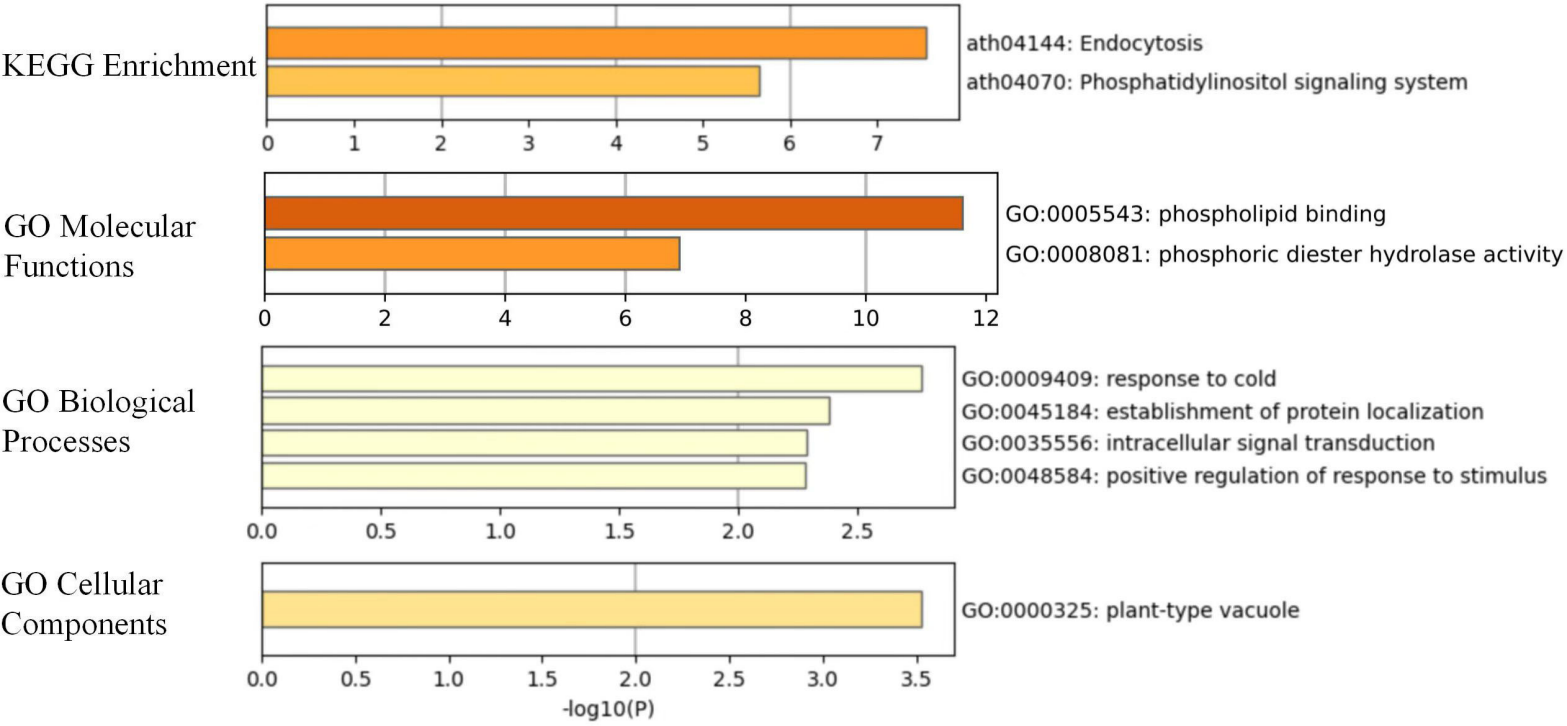
EGGand GO enrichment analysis of the *BrC2DP* gene family in Chinese cabbage.

To elucidate the potential biological functions of the *BrC2DP* gene family in Chinese cabbage, KEGG pathway enrichment and GO functional enrichment analyses were conducted in this study (**Figure 8**). Two significantly enriched KEGG pathways were screened out, in which the endocytosis pathway (ath04144) showed the most prominent enrichment, followed by the phosphatidylinositol signaling system (ath04070), implying that *BrC2DP* family members may be involved in intracellular endocytic transport and phosphoinositide-mediated signaling processes. GO enrichment analysis was further performed across three core functional categories. In the molecular function category, phospholipid binding (GO:0005543) was the most significantly enriched term, together with phosphoric diester hydrolase activity (GO:0008081), demonstrating that *BrC2DP* proteins possess potential functions in lipid binding and phosphodiester hydrolysis, which are closely related to membrane metabolism and signal transduction. Regarding biological processes, response to cold (GO:0009409) represented the top enriched item, followed by the establishment of protein localization (GO:0045184), intracellular signal transduction (GO:0035556), and positive regulation of response to stimulus (GO:0048584). These findings suggested that the *BrC2DP* gene family contributes to cold stress adaptation in Chinese cabbage, and also participates in the regulation of protein trafficking, intracellular signal transduction and stress response. For the cellular component category, only plant-type vacuole (GO:0000325) was significantly enriched, indicating that BrC2DP proteins are mainly localized to plant vacuoles.

## Discussion

*C2DPs* are widely present in eukaryotes and exert critical functions in signal transduction, membrane trafficking, and plant stress adaptation. The C2 domain is a canonical Ca^2+^-dependent phospholipid-binding module that mediates cellular signal transmission by interacting with membrane phospholipids (Nalefski and Falke, 1996; Chapman, 2002). Accumulating evidence has demonstrated that *C2DPs* perceive intracellular Ca^2+^ fluctuations and participate in signal cascades, thereby modulating plant growth, development, and responses to abiotic stresses including drought, salt, and low temperature (Sun et al., 2021; Zhang et al., 2022). Moreover, as a pivotal second messenger, Ca^2+^ plays a central role in plant adaptation to environmental stresses (Sanders et al., 2002).

### Expansion and evolutionary characteristics of the *BrC2DP* gene family

In this study, a total of 157 *BrC2DP* genes were identified in the *Brassica rapa* genome, indicative of a substantial expansion of this gene family in Chinese cabbage. Previous studies have characterized 82 *C2DP* genes in the rice genome (Zhang et al., 2022) and 180 C2 domain-encoding genes in soybean (Sun et al., 2021), revealing remarkable interspecific variations in the size of the *C2DP* gene family across plant species. Such variations are likely associated with gene duplication events and genome polyploidization events during plant evolution. *Brassica* species have undergone whole-genome triplication (WGT) throughout their evolutionary history, which is widely recognized as a major driving force for the expansion of gene families.

Chromosomal localization analysis revealed that *BrC2DP* genes are unevenly distributed across the 10 chromosomes of *Brassica rapa*, with distinct gene clusters formed in specific chromosomal regions. Analogous clustered distribution patterns have been reported for the *C2DP* gene families in rice and *Lotus corniculatus* (Zhang et al., 2022; Yang et al., 2025). Existing studies have proposed that tandem duplication and segmental duplication are core mechanisms underlying gene family expansion (Cannon et al., 2004). Collinearity analysis in the present work identified numerous collinear gene pairs, with a prominent enrichment on chromosomes A03 and A09, further confirming the essential contribution of gene duplication events to the evolution of the *BrC2DP* gene family.

Phylogenetic analysis partitioned the *BrC2DP* gene family into seven subgroups, a classification largely consistent with the phylogenetic architecture of the rice *C2DP* gene family (Zhang et al., 2022). Generally, genes within the same subgroup share conserved domain architectures and motif compositions, suggesting that these genes may derive from a common ancestral gene and have undergone functional divergence during long-term evolutionary processes.

### Potential roles of *BrC2DP* genes in drought stress response

Drought represents a major abiotic stress limiting plant growth and agricultural productivity. Plants sense environmental cues and orchestrate the expression of stress-related genes through intricate signal transduction networks, in which Ca^2+^ signaling acts as a core regulatory hub (Sanders et al., 2002). Upon exposure to drought or salt stress, intracellular Ca^2+^ concentrations undergo rapid dynamic changes, activating a suite of Ca^2+^-binding proteins and modulating downstream signaling pathways (Reddy et al., 2011). By virtue of their Ca^2+^-binding capacity and membrane-interacting properties, *C2DPs* function as critical mediators in stress signal transduction (Corbalan-Garcia and Gómez-Fernández, 2014).

Expression profiling in this study demonstrated that the majority of *BrC2DP* genes exhibited significant differential expression under drought stress, with a subset of genes showing persistent up-regulation or down-regulation. A comparable expression pattern has been reported in the soybean C2 domain gene family, where *GmC2–148* is significantly induced under both drought and salt stress conditions (Sun et al., 2021). Furthermore, studies have shown that *C2DPs* enhance plant stress tolerance by regulating antioxidant enzyme activities, osmoprotectant accumulation, and the transcription of stress-responsive genes (Zhu, 2016; Sun et al., 2021). These findings suggest that *BrC2DP* genes are likely involved in the regulatory networks governing drought stress responses in *Brassica rapa.*

Among all differentially expressed genes, *BrC2DP56* displayed the most dramatic up-regulation under drought stress, suggesting its pivotal role in plant drought response. A previous study in soybean reported that *GmC2–148* improves plant tolerance to drought and salt stresses by modulating the expression of multiple stress-responsive transcription factors, including *DREB*, *WRKY*, and *NAC* family members (Sun et al., 2021). These results further substantiate the essential roles of *C2DPs* in plant stress resistance regulatory networks.

### Functional significance of *BrC2DP56* in drought tolerance

Subcellular localization assays indicated that the BrC2DP56 protein is localized to the endoplasmic reticulum. Existing research has shown that C2DPs are typically distributed in membrane-associated compartments such as the plasma membrane, cytoplasm, or endoplasmic reticulum, and regulate plant stress responses via Ca^2+^-dependent signal transduction (Chapman, 2002; Corbalan-Garcia and Gómez-Fernández, 2014). For instance, the *Arabidopsis* SYT1 protein, a C2 domain-containing membrane protein, plays a vital role in maintaining plasma membrane stability and mediating plant adaptation to environmental stresses (Yamazaki et al., 2008). Functional characterization revealed that BrC2DP56 may act as a negative regulator of drought tolerance in *Brassica rapa*. Transgenic plants overexpressing BrC2DP56 exhibited more severe wilting under drought stress, whereas silencing of this gene enhanced drought resistance in Chinese cabbage. While most *C2DPs* are documented as positive regulators of stress tolerance, several family members function as negative regulators in signaling networks, which may be attributed to complex feedback regulatory mechanisms or crosstalk between distinct signaling pathways (Niu et al., 2022).

## Data Availability

Publicly available datasets were analyzed in this study. This data can be found here: http://brassicadb.cn.
