# Supplementary figures and images for: Identification of the BrC2DP gene family in Chinese Cabbage and functional analysis of the BrC2DP56 gene in drought stress
Source: Front Plant Sci. 2026 May 8;17:1839463. doi: 10.3389/fpls.2026.1839463 (PMC13195685; doi:10.3389/fpls.2026.1839463)

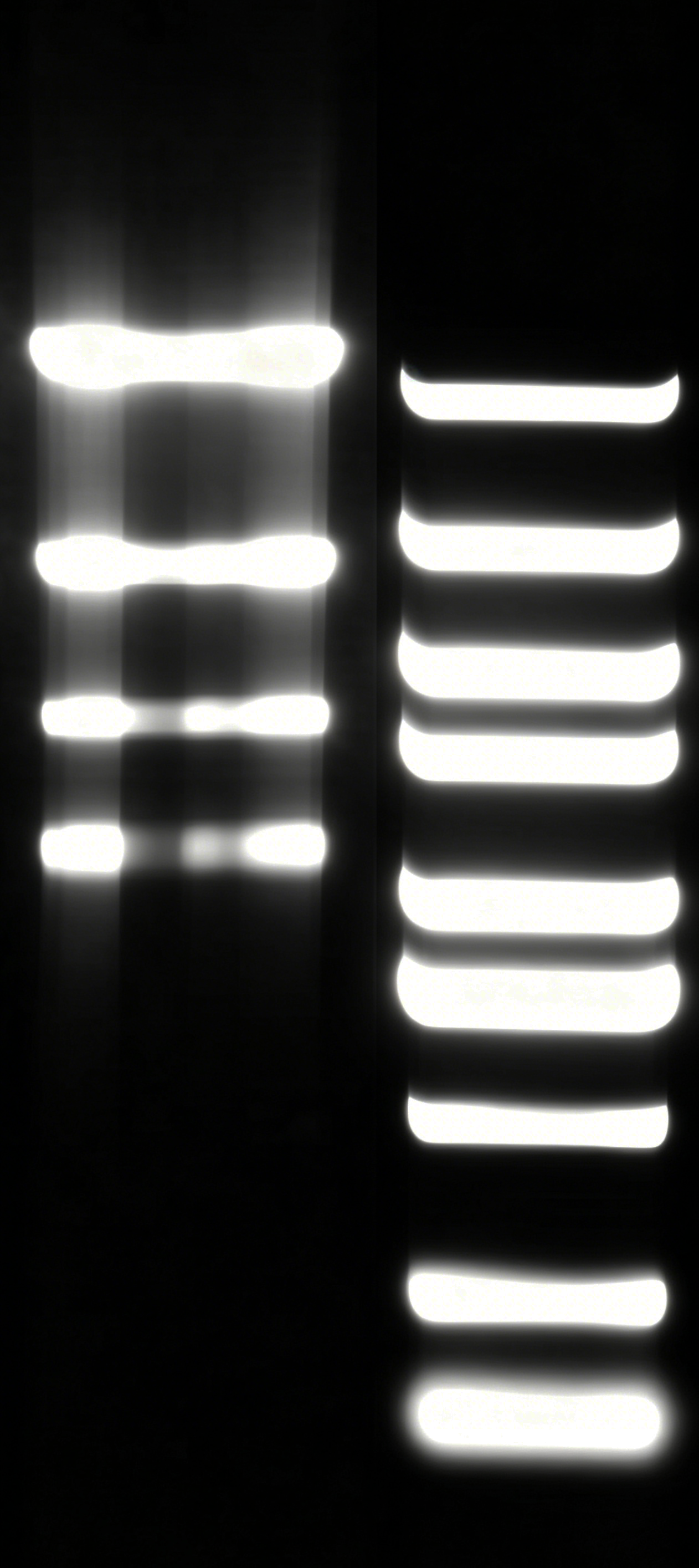

Supplement: Supplementary file 1 [file DataSheet1.zip › Supplementary materials/BrC2DP56 transient overexpression vector gel image.png]

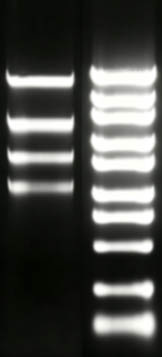

Supplement: Supplementary file 1 [file DataSheet1.zip › Supplementary materials/BrC2DP56 VIGS vector gel image.jpg]

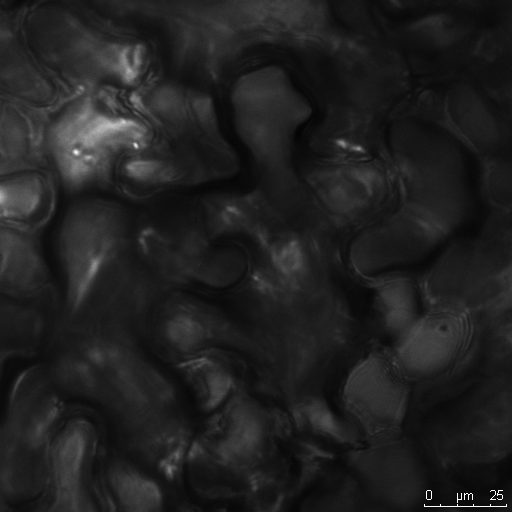

Supplement: Supplementary file 1 [file DataSheet1.zip › Supplementary materials/BrC2DP56-GFP-Bright.tif]

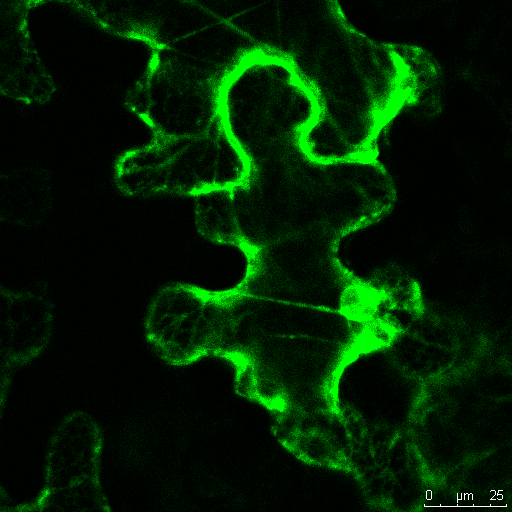

Supplement: Supplementary file 1 [file DataSheet1.zip › Supplementary materials/BrC2DP56-GFP-GFP.tif]

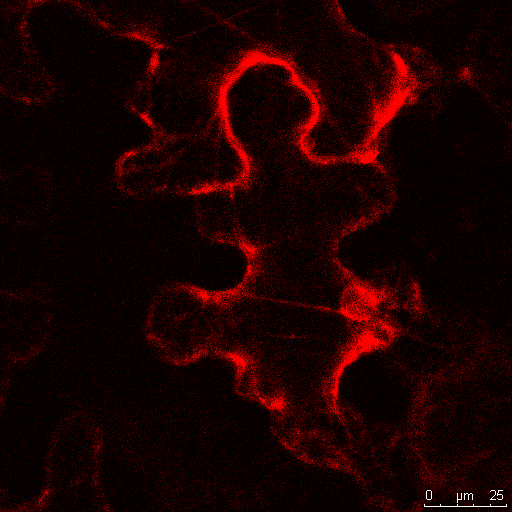

Supplement: Supplementary file 1 [file DataSheet1.zip › Supplementary materials/BrC2DP56-GFP-HDEL-mCherry.tif]

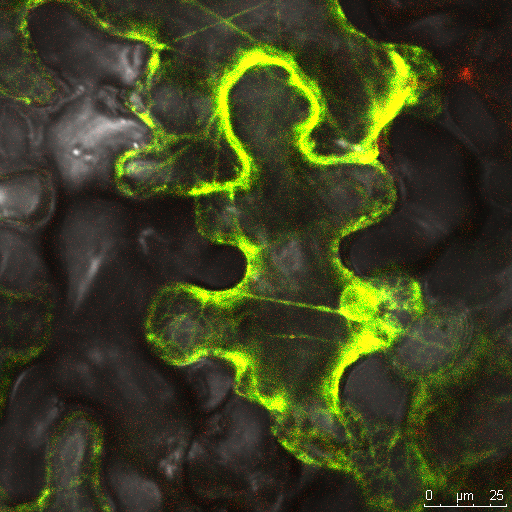

Supplement: Supplementary file 1 [file DataSheet1.zip › Supplementary materials/BrC2DP56-GFP-Merge.tif]

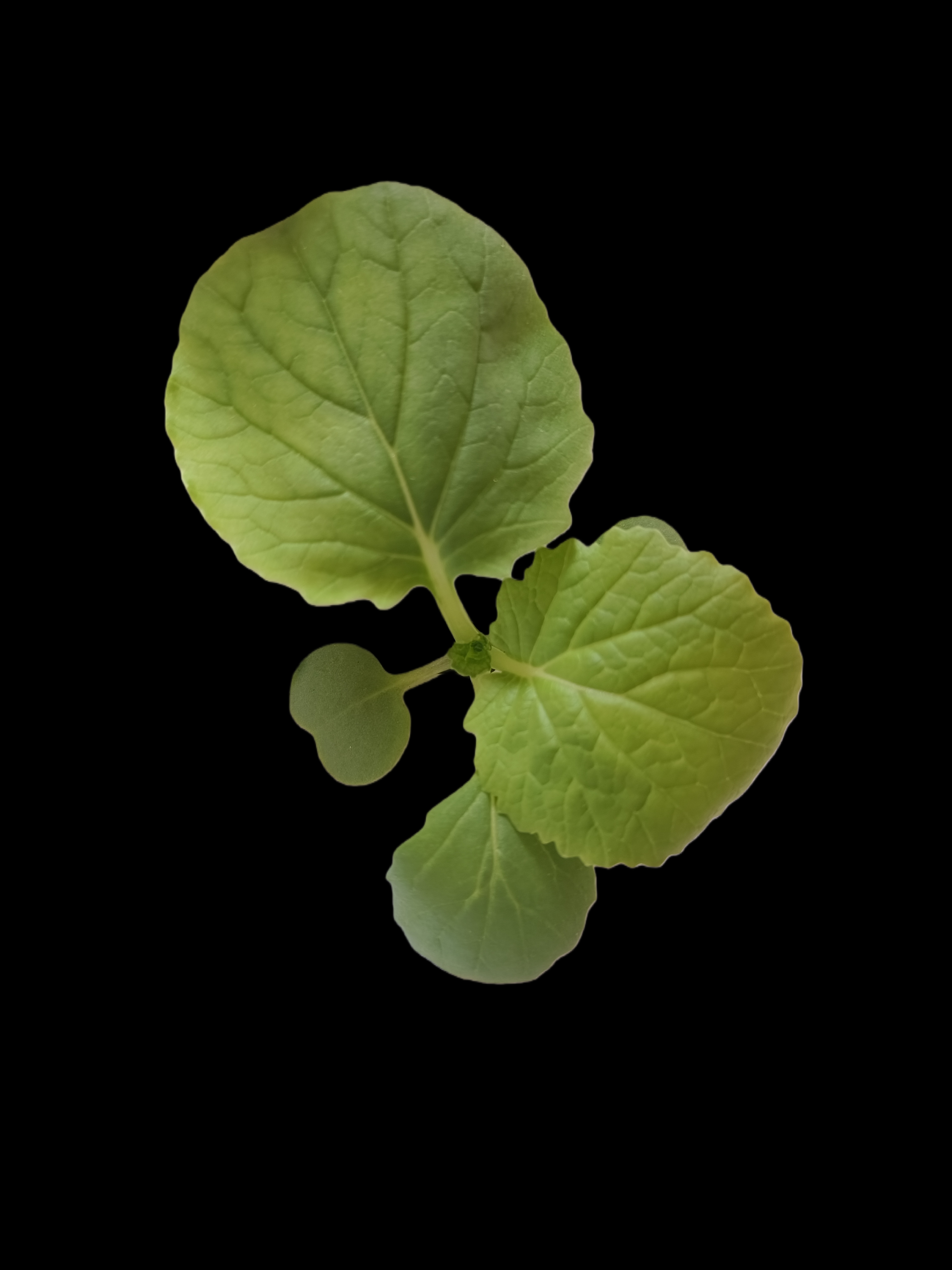

Supplement: Supplementary file 1 [file DataSheet1.zip › Supplementary materials/CK.jpg]

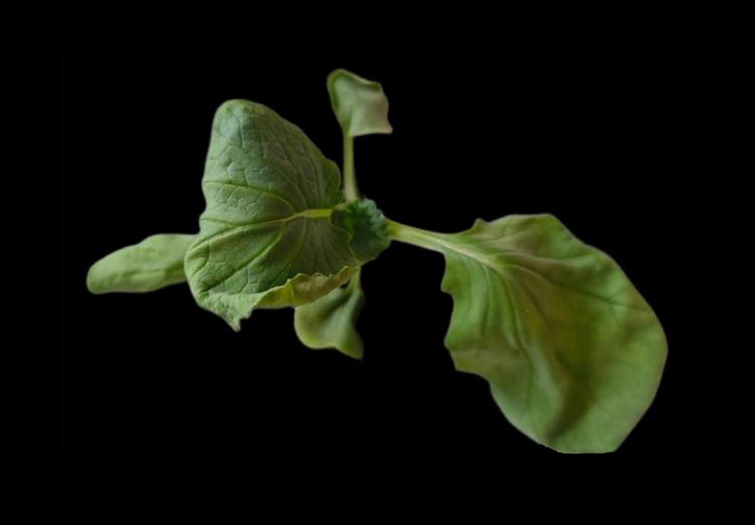

Supplement: Supplementary file 1 [file DataSheet1.zip › Supplementary materials/Drought-pSuper BrC2DP56.jpg]

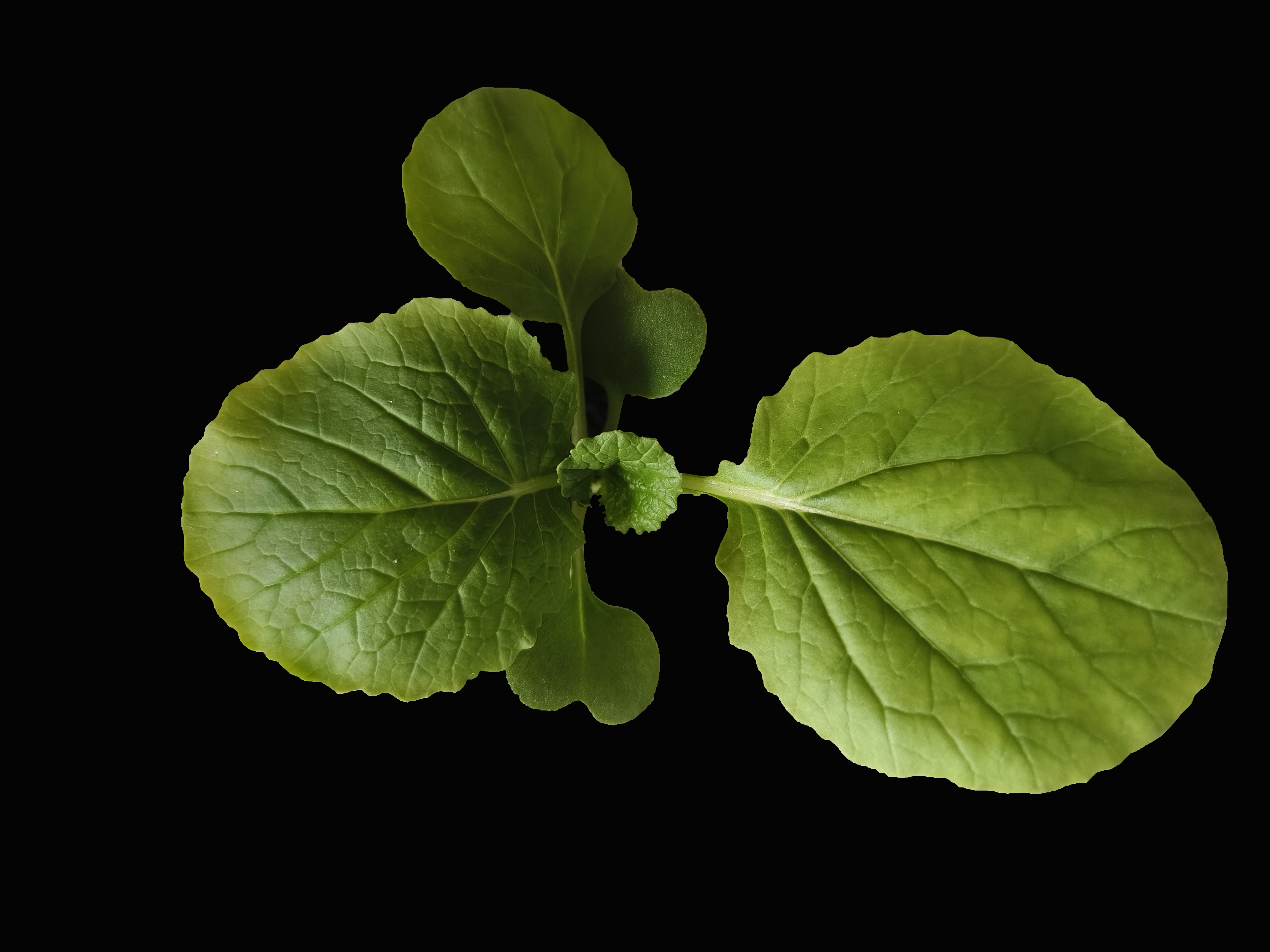

Supplement: Supplementary file 1 [file DataSheet1.zip › Supplementary materials/Drought-TRV BrC2DP56.jpg]

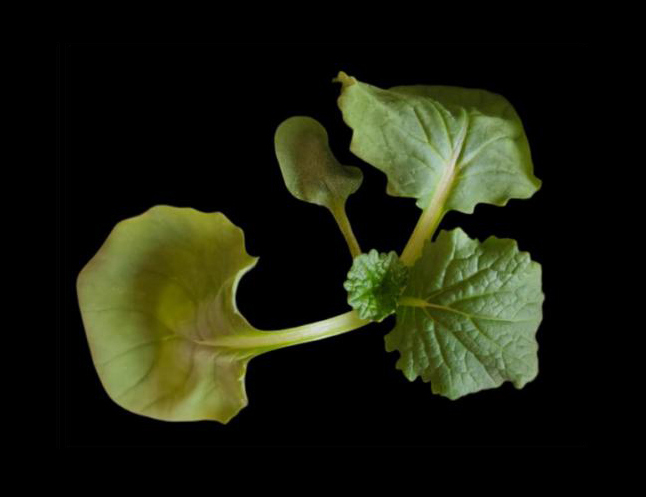

Supplement: Supplementary file 1 [file DataSheet1.zip › Supplementary materials/Drought-WT.jpg]

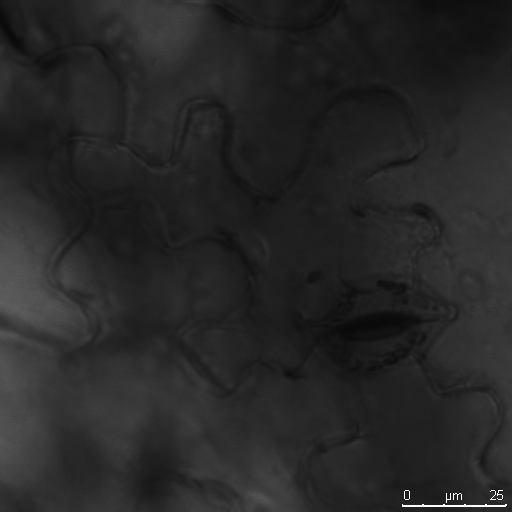

Supplement: Supplementary file 1 [file DataSheet1.zip › Supplementary materials/Free GFP-Bright.tif]

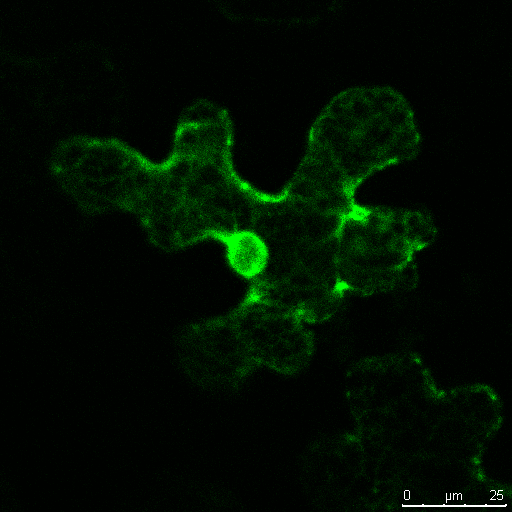

Supplement: Supplementary file 1 [file DataSheet1.zip › Supplementary materials/Free GFP-GFP.tif]

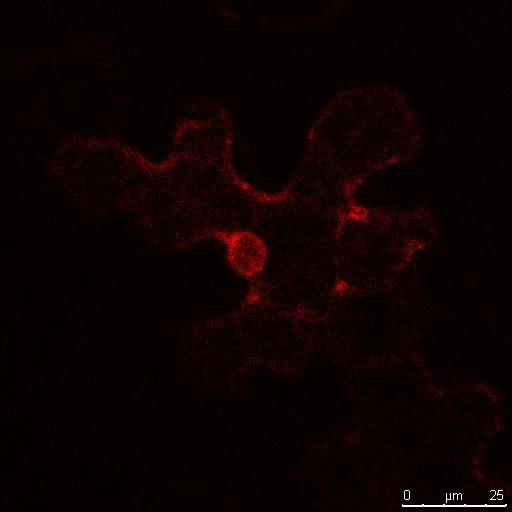

Supplement: Supplementary file 1 [file DataSheet1.zip › Supplementary materials/Free GFP-HDEL-mCherry.tif]

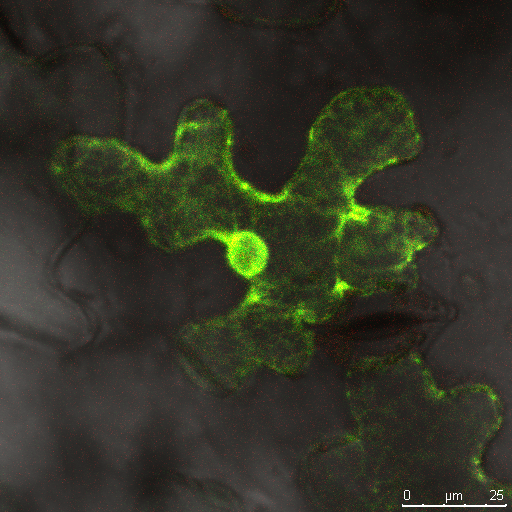

Supplement: Supplementary file 1 [file DataSheet1.zip › Supplementary materials/Free GFP-Merge.tif]
